# BO-ALLCNN: Bayesian-Based Optimized CNN for Acute Lymphoblastic Leukemia Detection in Microscopic Blood Smear Images

**DOI:** 10.3390/s22155520

**Published:** 2022-07-24

**Authors:** Ghada Atteia, Amel A. Alhussan, Nagwan Abdel Samee

**Affiliations:** 1 Department of Information Technology, College of Computer and Information Sciences, Princess Nourah Bint Abdulrahman University, P.O. Box 84428, Riyadh 11671, Saudi Arabia; geatteiaallah@pnu.edu.sa (G.A.); nmabdelsamee@pnu.edu.sa (N.A.S.); 2 Department of Computer Sciences, College of Computer and Information Sciences, Princess Nourah Bint Abdulrahman University, P.O. Box 84428, Riyadh 11671, Saudi Arabia

**Keywords:** leukemia, Bayesian optimization, convolutional neural network, CNN, deep learning, classification, hyperparameter optimization

## Abstract

Acute lymphoblastic leukemia (ALL) is a deadly cancer characterized by aberrant accumulation of immature lymphocytes in the blood or bone marrow. Effective treatment of ALL is strongly associated with the early diagnosis of the disease. Current practice for initial ALL diagnosis is performed through manual evaluation of stained blood smear microscopy images, which is a time-consuming and error-prone process. Deep learning-based human-centric biomedical diagnosis has recently emerged as a powerful tool for assisting physicians in making medical decisions. Therefore, numerous computer-aided diagnostic systems have been developed to autonomously identify ALL in blood images. In this study, a new Bayesian-based optimized convolutional neural network (CNN) is introduced for the detection of ALL in microscopic smear images. To promote classification performance, the architecture of the proposed CNN and its hyperparameters are customized to input data through the Bayesian optimization approach. The Bayesian optimization technique adopts an informed iterative procedure to search the hyperparameter space for the optimal set of network hyperparameters that minimizes an objective error function. The proposed CNN is trained and validated using a hybrid dataset which is formed by integrating two public ALL datasets. Data augmentation has been adopted to further supplement the hybrid image set to boost classification performance. The Bayesian search-derived optimal CNN model recorded an improved performance of image-based ALL classification on test set. The findings of this study reveal the superiority of the proposed Bayesian-optimized CNN over other optimized deep learning ALL classification models.

## 1. Introduction

Leukemia is a fatal malignancy that attacks blood cells. It is prevalent in children as well as adults aged 65 and beyond [1]. Leukemia is among the types of cancers that occurs with great frequency in the United States (US) along with lung, colorectal, breast, and prostate cancers [1]. The Surveillance, Epidemiology, and End Results (SEER) Program, which is responsible for providing cancer statistics in US, estimates that 60,650 new cases of leukemia will be diagnosed and 24,000 individuals will die from this disease in the United States in 2022 [2]. Leukemia originates in bone marrow and may spread to other organs via the bloodstream. Red blood cells, white blood cells (WBCs), and platelets are produced by bone marrow, which is found in the interior bone cavity. Blood cells are derived from stem cells that develop into lymphoid and myeloid cells as they mature and are subsequently discharged into the bloodstream [3]. Leukemia occurs when myeloid or lymphoid cells begin to grow fast and uncontrollably. The formation of such a large number of defective leukemia cells diminishes the likelihood of normal blood cell development in the bone marrow. Inadequate amounts of healthy blood cells discharged into the bloodstream result in insufficient oxygen supply to the organs, impaired blood clotting, and a weakened immune system’s capacity to combat infections.

Acute Lymphocytic Leukemia, or ALL, is the most prevalent form of leukemia, in which lymphoid cells proliferate abnormally in the bone marrow. ALL primarily affects children and adolescents. Leukemia, unlike other types of cancer, does not produce a tumor that could be detectable by medical imaging. Therefore, leukemia is identified using further medical procedures, such as complete blood count, lumbar punctures, myelograms, and bone marrow biopsies [4]. Initially, leukemia patients are screened through the microscopic examination of blood smears on glass slides. The examination of microscopic slides and the diagnosis of diseases are carried out by domain-specialists. This procedure is time-consuming, laborious, and depends on the operator’s experience. Due to the rapid development and progression of ALL over a short period of time, early detection is essential for the prompt treatment of this lethal disease. Automated diagnosis approaches are urgently required to reduce human intervention in the inspection process, expedite the process, and increase the accuracy of leukemia detection.

Image classification algorithms have been used to detect blood cancer in medical images in recent years. There are two types of classification approaches for leukemia diagnosis [4]: standard machine learning (ML); and deep learning (DL) [5,6]. Preprocessing images for object enhancement and segmentation is a common practice in the traditional ML approach. Conventional classifiers such as KNN, Naive Bayesian (NB), SVM, and neural networks [7,8,9,10,11,12] have been used to extract distinguishing features from the images. It is true that the performance of classical ML methods is generally acceptable, but this is highly dependent on many factors. The characteristics of the datasets, the effectiveness of image enhancement processes, the accuracy of the segmentation algorithm used and the quality of extracted features, and the structure of the ML classifier itself are factors that significantly affect the performance of ML models [6,13,14]. To address these limitations, recent research has focused on employing deep learning neural networks in the diagnosis of various diseases [9,15].

In recent years, a huge interest in deep learning has grown [16]. It has been utilized in several life aspects [17,18]. Convolutional neural network (CNN) is the most established algorithm among various deep learning models. CNN is a class of artificial neural network that has dominated computer vision tasks and reached expert-level proficiency in numerous domains including computerized medical diagnosis [9,19]. CNN-based computer-aided diagnosis (CAD) systems have been recognized for their effectiveness in detecting the presence of numerous diseases, such as diabetic retinopathy and its complications [20,21], various types of cancer [22,23], and COVID-19 [12]. This cutting-edge technology has been used for image segmentation [24,25,26,27], classification [28,29], and object identification and recognition [30,31,32]. The CNN structure is designed to learn spatial hierarchies of features automatically and adaptively from gridded data such as images [19]. To fulfill this task, CNN contains a tremendous number of learnable parameters, network weights, and biases, to be tuned during the training process using labeled data. Network parameters are tuned by minimizing the difference between network output and data labels through the backpropagation algorithm [33]. This process is performed during training through an optimization algorithm such as the Gradient Decent, Stochastic Gradient Decent, Adaptive Moment Estimation, and others [33]. In order to govern the training process of the model, another set of variables, called model hyperparameters, should be set before training the network.

Model hyperparameters are points of configuration of the learning process that allow a machine learning model to be customized for a specific task and dataset [34]. Hyperparameters include learning rate, number of epochs, number of hidden layers in a neural network, specific settings of the parameters optimization algorithm, the number of hidden neurons, activations functions, and others. Hyperparameters directly impact the training algorithm’s behavior and they substantially affect the performance of the trained model [34]. Manual tuning of hyperparameters, although widely used in the literature, is not considered the best practice to help guide the learning process. Tuning hyperparameters is a critical step in developing vigorous predictive models. Nevertheless, the selection of the best combination of interacting hyperparameters for a given dataset is challenging. This process is called hyperparameters optimization. The optimization procedure involves defining a search space for the hyperparameters. In this space, each hyperparameter represents a unique dimension and each point in the space corresponds to a vector of all hyperparameters’ values that is related to one configuration of the ML model [28]. The optimization procedure aims to find the set of hyperparameters that provides the best performance of the ML model through an iterative procedure. There are popular approaches for configuring hyperparameters such as the Grid Search and Random Search. These approaches are uninformed search methods in the sense that they treat search iterations independently [34]. The algorithm does not make use of previous iterations in the choice of the set of hyperparameters to be used in the current iteration. The Grid Search method examines all unique combinations of hyperparameters in the search space to declare the combination that provides the best prediction performance. This approach is simple but suffers from high computational time especially for increased search space size. The Random Search approach evaluates a predetermined number of hyperparameter sets at random. This strategy reduces run time but could miss the optimal set of hyperparameters supplying best model performance. A more advanced search method is the Bayesian optimization. Bayesian optimization, unlike the aforementioned search methods, is an informed search approach that utilizes knowledge from previous iterations to select forthcoming sets of hyperparameters [34]. It compromises between moderate run time and search efficiency to provide an optimal set of hyperparameters that provides the best performance of the ML model. 

In this study, we propose a customized CNN that has been trained from scratch to detect the presence of Acute Lymphoblastic Leukemia in a hybrid set of microscopic blood smear images. The Bayesian optimization approach is utilized to determine the optimal CNN architecture and optimize the hyperparameters of the proposed deep network. Optimizing the architecture and hyperparameters of the proposed deep learning network would improve the capability of distinguishing between normal and diseased blood images, increase the classification performance, and thus provide a robust automatic ALL-CAD system. The main contributions of this study are listed as follows:

1. Development of a new optimized deep learning CNN for the detection of ALL in microscopic blood smear images.
2. Formation of a hybrid dataset by integrating two public ALL datasets to supplement the input data.
3. Augmentation of the hybrid dataset using several image transformations to promote classification performance.
4. Determination of the optimal architecture and hyperparameters of the proposed CNN using the Bayesian optimization approach.
5. Comparison between the optimized model and a non-optimized model to investigate the effectiveness of Bayesian optimization in improving classification performance.
6. Classification performance comparison of the optimal ALL CNN model with that of the state of the art.

The paper is organized as follows: Section 2 presents a review of the research conducted for leukemia detection. Section 3 provides a description of the used dataset, the proposed framework, and methodologies. Section 4 presents the study findings and results discussion, and Section 5 concludes the work.

## 2. Literature Review

Automatic diagnosis of leukemia has become crucial in accelerating the detection of such a deadly disease. In response to this demand, many studies have tackled the problem of leukemia detection in blood smear images using diverse methodologies. Many classical machine learning tools have been utilized to classify leukemia in blood images. For instance, Negm et.al [35] developed a decision support system for the diagnosis of acute leukemia blast cells in colored microscopic images. This system segmented leukemia cells and extracted their features using K-means and then classified the cells according to their morphological features. The system was tested using a private dataset and recorded an accuracy of 99.5%, sensitivity of 99.3%, and specificity of 99.5%. Begum et al. [36] proposed a leukemia diagnosis system that utilizes Hybrid Fuzzy C-Means with Cluster Center Estimation and an SVM classifier. The Hybrid Fuzzy C-Means with Cluster Center Estimation algorithm was used to separate the nuclei of blood cells. Morphological operations were then applied to extract the geometric features of the nuclei. Extracted features were then fed to an SVM algorithm to classify the cell image into normal or malignant cell. That system achieved an accuracy of 83% on a private dataset. Jothi et al. [37] extracted five types of features from segmented nucleus images of WBC images and used them with a number of classical ML classifiers for the differentiation of ALL cells from healthy lymphocytes. The extracted features were the texture, color, morphological, wavelet, and statistical features. They used three types of hybrid supervised feature selection methods for selecting these features. Classification algorithms such as Naïve Bayes, linear discriminant analysis, *K*-nearest neighbor, support vector machine, decision tree, and ensemble random under-sampling boost were applied on a leukemia dataset. The authors applied Jaya algorithm to optimize the rules generated from the classification algorithms. Their findings showed that the classification accuracy of the used classifiers was improved after optimizing with the Jaya algorithm. Agaian et al. [38] presented a simple technique for the automatic segmentation and detection of nucleated cells to diagnose Adult Acute Myeloid Leukemia (AML) in blood smear images. They utilized the local binary pattern algorithm and compared the Hausdorff dimension on the system to classify the input image. By testing their system on eighty microscopic blood images, their system achieved an accuracy of 98%. Another system was developed by Kazemi et al. [39] to detect AML and its subtypes in microscopic blood images. White blood cells were segmented from multi-cell smear images and multiple features were extracted from the segmented cell nuclei. The color, texture, shape, Hausdorff dimension, irregularity, and nucleus–cytoplasm ratio were extracted from the nuclei of the white blood cells and found to be the most distinguishing features. The SVM classifier was used to classify the input images into cancerous and noncancerous as well as to classify the cancerous images into their subtypes. Their results showed that the algorithm achieved a classification accuracy of 96% for the binary classification and 87% for the AML subtypes classification, respectively. Another study [40] developed a system to diagnose ALL and its subtypes using SVM algorithm. They employed the K-means clustering for segmenting the nuclei of the blood cells. Some statistical and geometric features were extracted from the segmented nuclei and fed to an SVM classifier. Their system achieved a sensitivity of 98%, specificity of 95%, and accuracy of 97% for the binary classification of ALL. However, the system recorded 84.3%, 97.3%, and 95.6%, for the sensitivity, specificity, and accuracy, respectively, for the subtype classification. In a neural network-based system developed by Muthumayil et al. [41] to diagnose chronic lymphocytic leukemia in WBC images, the Enhanced Color Co-Occurrence Matrix algorithm was used to extract the features from the blood images and an Enhanced Virtual Neural Network was developed for the classification of the input images. The values recorded for that study are 97.8% for sensitivity, 89.9% for specificity, and 76.6% for accuracy. In another study [42], artificial neural network (ANN) was used to automatically segment leukocyte cells in microscopic blood images, where the characteristics of white blood cells were extracted using the neighboring pixel algorithm. The most distinguishing statistical features were selected using the Genetic Algorithm. This technique yielded an accuracy of 97% for blast cell segmentation. The work of Bodzas et al. [43] proposed a system to automatically identify ALL in blood smear images by introducing a three-phase filtration algorithm to enhance the smear images followed by a segmentation step. The classification was performed using two ML algorithms: ANN and SVM. Their findings showed that both classifiers recorded a specificity of 95.3% and sensitivity of 98.2% and 100% for the SVM and the ANN, respectively.

With the evolution of deep learning technologies, many deep learning-based CAD systems have been developed to detect leukemia in blood images. Eckardt et al. [20] applied a multi-step deep learning approach to segment cells from bone marrow images to distinguish between AML and healthy cells and predict the status of the most common mutation in AML named as Nucleophosmin 1. In that work, the segmentation is performed in two steps. Initially, the VGG Image Annotator was used to segment the marrow images. After that, Faster Region-based Convolutional Neural Network (FRCNN) was trained using the segmented images. After hematologists refined the images manually, the final segmentation was then declared. They obtained the area under the receiver operating characteristic of 0.97 and 0.92, for the AML segmentation and the mutation prediction, respectively. Although the findings of this study are promising, it requires human intervention which makes it prone to errors. Fan et al. [24] developed a deep convolutional neural network to localize and segment leukocytes in microscopic blood images. Information from the image pixels was used to train the CNN in a supervised manner. The trained CNN was used to locate the region of interest of the leukocyte and generate the segmentation mask. Their findings showed that the introduced CNN is robust and achieves competitive segmentation accuracy. Ouyang et al. [30] proposed a diagnosis system for AML based on instance segmentation with CNNs. A custom dataset of microscopic blood images were subjected to instance segmentation using the Mask R-CNN algorithm to detect the nucleated WBCs. A deep learning neural network was created and trained from scratch using the segmented images. The authors compared the performance of their system when trained by the custom dataset with the performance of the network when pretrained on the MS Coco dataset and fine-tuned it with the custom dataset. The study results showed that the pretrained model outperformed the model trained from scratch in terms of average precision.

Various pretrained CNNs have been developed recently and used for automatic classification of medical images. Pretrained CNNs such as GoogleNet, AlexNet, Inception, and others are deep networks that were trained using the enormously huge dataset, ImageNet dataset. Pretrained CNNs have the capability to classify images efficiently through transfer learning. Transfer learning enables the classification of a specific dataset by fine-tuning the final layers of the pretrained CNN [44,45]. Shafique et al. [46] created a technique for automatically diagnosing ALL and its three subtypes (L1, L2, and L3) using blood smear images. They modified the pre-trained AlexNet CNN by altering the final layers in order to classify input photos into one of four categories. The tuned AlexNet achieved an accuracy of 99.5%, recall of 100%, and specificity of 98.1% for ALL binary classification. However, for subtype categorization, their method had a sensitivity of 96.7%, a specificity of 99%, and an accuracy of 96%. Loey et al. [7] utilized two deep learning classification models to diagnose the presence of leukemia in microscopic images of blood. In the first model, the pretrained AlexNet CNN was used to extract the features from the input images, and they used a number of classical ML classifiers. The used classifiers were the SVMs, linear discriminants (LDs), decision trees (DTs), and KNNs. In the second model, transfer learning was employed for classification. In the transfer learning approach, the final layers of the AlexNet CNN were fine-tuned to suite the problem in hand. The image dataset was preprocessed and augmented prior to extracting the features. The study results showed that for the first model, the SVM classifier achieved the best accuracy of 99.79%. It has also been shown that the transfer learning classification with 10-fold cross-validation yielded an accuracy of 99.82%. The research conducted by Bibi et al. [10] developed an Internet of Medical Things (IoMT) framework that leveraged cloud computing and clinical devices to facilitate real-time coordination for the diagnosis and treatment of leukemia. In the ALL-IDB and ASH image bank datasets, the Dense Convolutional Neural Network (DenseNet-121) and Residual Convolutional Neural Network (ResNet-34) were utilized to identify leukemia subtypes. Transfer learning was used to carry out classification by altering the structure of pretrained CNNs. The results indicated that the subtype classification accuracy for the DenseNet and ResNet models was 99.1% and 99.5%, respectively. Mondal et al. [47] presented a system for the diagnosis of ALL that consists of a weighted ensemble of deep CNNs. Xception, VGG-16, DenseNet-121, MobileNet, and InceptionResNet-V models were used to build the ensemble. Using the F1-score, area under the curve (AUC), and Kappa values of the relevant CNN, the model weights were computed. The findings of this investigation indicated that the weighted ensemble model achieved an F1-score of 89.7%, an accuracy of 88.3%, and an AUC of 0.948. The study concluded that the ensemble model yields a better classification performance than that of the individual CNN components. 

The aforementioned studies and others have provided encouraging results for the use of deep learning technologies in diagnosing leukemia. However, few studies have focused on enhancing the performance of deep learning-based leukemia classifiers through optimization approaches. Hyperparameter tuning and feature selection are two effectual factors in deep learning networks’ performance. Optimization algorithms have been proven to be effective tools for selecting optimal features as well as optimizing hyperparameters of ML models in several applications [48]. Recent methods used for CNN structure optimization in the medical field include the hybrid sine–cosine algorithm [49], chimp optimization algorithm [50], and Whale Optimization [51]. Some recent studies used several optimization approaches to select optimal features for leukemia classification problems [52,53,54,55,56]. For instance, Abdeldaim et al. [53] used the bio-inspired Salp Swarm Optimization Algorithm to select the optimal features from the classification of ALL using several classical ML classifiers. Features extracted by the pretrained VGGNet were filtered using the bio-inspired Salp Swarm optimization Algorithm before the classification of the WBCs. The optimization algorithm selected 1000 out of 25,000 features and these features were fed to a number of classical classifiers including the KNN, SVM, Decision Trees, Naive Bayes, and others. The best accuracy obtained using this methodology was 96.11%. Another work [54] employed the Social Spider Optimization Algorithm (SSOA) to select the most appropriate features for ALL detection in the ALL-IDB2 dataset. The WBCs were first segmented from the images and several features such as color, shape, texture, and hybrid features were extracted from segmented cells. Afterwards, the best features were selected using SSOA and fed to several classical ML classifiers. The results showed that the classification accuracy of their proposed model was 95.23% which was higher than that of non-SSOA. 

Pertaining the use of optimization algorithms for tuning hyperparameters of deep learning models, very few studies have been found to tackle this problem. For instance, Ramaya et al. [52] developed an enhanced deep CNN with Arithmetic Optimization algorithm for the detection of AML from the Munich AML Morphology Database. They preprocessed the images for noise removal, then segmented the cell nucleus using the Modified Distance Regularized Level Set Evolution algorithm. After that the classification is carried out by a deep CNN. They used the Arithmetic Optimization algorithm to improve the classification performance of the CNN and obtained a classification accuracy of 99.82%. In another work by Praveena et al. [55], an automatic method for detecting ALL was proposed. This method was based on deep CNN that was trained using the Grey Wolf-based Jaya optimization algorithm. The input blood smear image was preprocessed, and WBCs were segmented using the Sparse Fuzzy C-Means clustering algorithm. Afterwards, the Local Directional Patterns and the color histogram-based features were extracted from the segmented images. The extracted features were fed to the CNN for the classification. On the ALL-IDB2 database, the introduced methods recorded 93.5% for accuracy, 95.3% for the sensitivity, and 93.9% for the specificity, respectively. The work of Hamza et al. [56] utilized the competitive swarm optimization (CSO) algorithm to optimize the hyperparameters of an attention-based long-short term memory model for the identification of ALL. They used the EfficientNetB0 algorithm to extract and select features from smear images after noise filtering and segmentation. Their approach recorded a classification accuracy of 96%. It is noticeable that the topic of hyperparameter optimization of deep learning-based leukemia detectors has not received considerable attention in the literature. There is a lack of studies that investigate the effectiveness of using various optimization algorithms for DL network hyperparameter tuning for leukemia detection. In response to this awareness, in this study, a new optimized convolutional neural network for Acute Lymphoblastic Leukemia detection in microscopic blood smear images is proposed. The Bayesian optimization algorithm is utilized to optimize the architecture of the proposed CNN and find the optimal set of network hyperparameters. The effectiveness of using Bayesian optimization for hyperparameter tuning on the classification performance of the proposed network is also investigated in comparison to recent approaches.

## 3. Materials and Methods

### 3.1. Datasets

Colored blood microscopic images from the ALL-IDB datasets [57] are used in this work. The ALL-IDB dataset includes two subsets, namely the ALL-IDB1 and the ALL-IDB2. Images of the ALL-IDB1 contains multiple cells while the ALL-IDB2 images presents a single cell at the slide center. Images in the dataset are sorted into two classes, healthy and diseased. Healthy images are labeled as the negative class and ALL-diseased images are labeled as the positive class by expert oncologists. The ALL-IDB1 subset is composed of 59 healthy images and 49 diseased images with a total of 108 images. ALL-IDB2 subset has a total of 260 images with 130 images in each of the two classes. In the current study, as we train a deep learning model, a hybrid dataset of blood smear images is constructed by fusing the two subsets together to supplement the data. The hybrid dataset contains a total number of 368 blood smear images with 189 healthy images and 179 ALL images.

### 3.2. Proposed Framework

**Figure 1:**
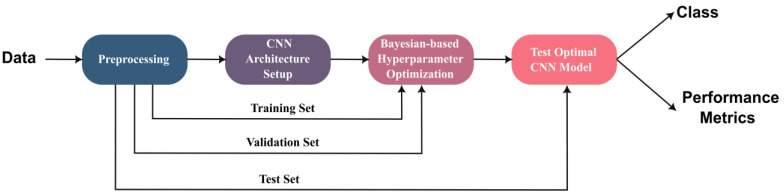
Framework of the proposed study.

The framework proposed in this study for ALL detection is presented in Figure 1. The first stage of this framework is preprocessing the image dataset. In the second stage, the architecture of the proposed deep learning network is set up. Network hyperparameter optimization is then conducted followed by testing the optimum network. A detailed description of the framework is provided in this section.

#### 3.2.1. Data Preprocessing

**Figure 2:**
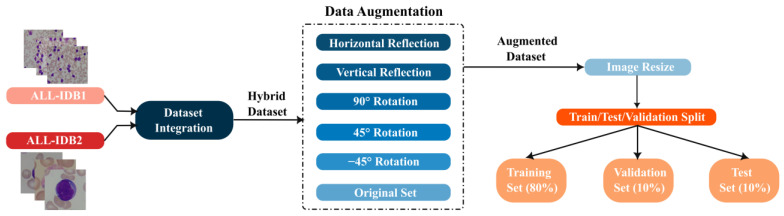
Preprocessing procedures applied to the input ALL-IDB datasets.

The RGB images from the ALL-IDB datasets are utilized to detect ALL in this study. The image data undergoes preprocessing procedures for hybrid set generation, augmentation, resizing, and splitting as illustrated in Figure 2. 

**Figure 3:**
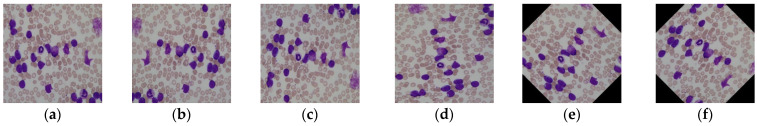
Sample of augmented microscopic blood images; (**a**) original; (**b**) vertically reflected; (**c**) horizontally reflected; (**d**) 90° rotated; (**e**) 45° rotated; (**f**) −45° rotated.

As aforementioned, a hybrid dataset is generated by integrating the ALL-IDB1 and the ALL-IDB2 to increase the size of the input dataset. Nonetheless, the hybrid dataset’s number of images is still considered too small to provide acceptable performance from the deep learning network. Therefore, in the current study, a data augmentation strategy is applied to extend the input dataset. Dataset augmentation has been found to reduce overfitting and improve the generalization capabilities of deep learning networks, as well as promoting classification performance [10]. Image cropping, translation, mirroring, and rotation are effective manipulations for augmenting image datasets. To augment the dataset in the present study, horizontal and vertical reflections, and image rotation with $\pm 45^{{}^{\circ}}$ and $90^{{}^{\circ}}\;$of all images in the hybrid dataset are utilized. Figure 3 displays a sample of the augmented images of an original smear image and its corresponding transformations. The enlarged dataset is formed by integrating the original and transformed images. A total of 2208 images with 1134 healthy images and 1074 ALL-diseased images made up the augmented dataset. The RGB channels of the augmented dataset are resized to [64 × 64] pixels to accommodate the proposed CNN input layer size. The dataset is then split into three sections: 80% for training, 10% for validation, and 10% for testing. The training set is composed of 1768 images while each of the validation and testing sets consists of 220 images. 

#### 3.2.2. Proposed CNN Architecture Setup 

Convolutional neural networks are proficient deep learning tools that have been used widely for image classification. CNNs are superior to classical neural networks in providing better prediction performance while maintaining fewer network parameters. A CNN is composed of several layers: convolutional layers, max-pooling/average-pooling layers, and fully-connected layers. This structure of CNN enables two roles: feature extraction and classification. Convolutional layers perform feature extraction, which is the process of obtaining distinguishing attributes from input images. To execute the classification task, feature maps created by convolutional layers are supplied to a fully connected layer. In this paper, we present a customized CNN architecture that is trained from scratch. The proposed CNN is designed to have three convolutional blocks. Each block is comprised of a number of convolution layers, batch normalization layers, and ReLu layers, followed by a pooling layer. These blocks are used to extract features. The final three layers, the average pooling layer, fully connected layer, and softmax layer, perform image classification. The number of convolutional layers per block is determined based on an optimized classification performance. Bayesian optimization is applied to optimize the hyperparameters of the proposed network and determine the optimal structure of the proposed CNN.

#### 3.2.3. Hyperparameter Optimization 

**Figure 4:**
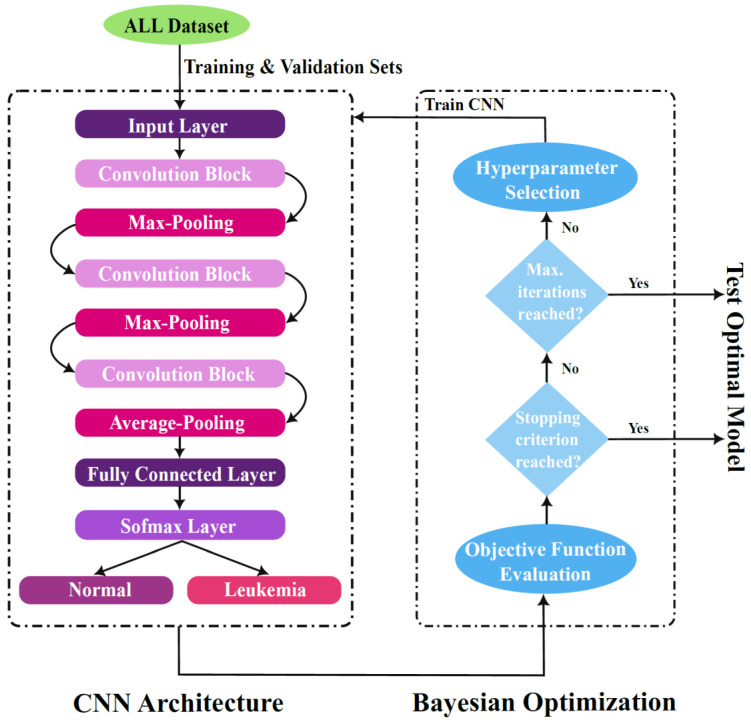
Proposed CNN architecture and Bayesian Optimization for Hyperparameter tuning.

Machine learning models have parameters and hyperparameters. Model parameters are the internal coefficients of the ML model. For instance, in a neural network, the model parameters are the neurons’ weights and biases. Parameters are learned automatically during training the model on a training dataset. On the other hand, model hyperparameters are the properties that control the whole training process. Hyperparameters can either be set manually by the developer or tuned through an optimization algorithm. The Bayesian optimization approach is used in this study to find the optimal design of the proposed deep neural network and optimize its hyperparameters. In this stage, network hyperparameters to be optimized are determined, objective function is created, and optimization is conducted. This stage selects the optimal model which is then tested using the holdout test set. Figure 4 demonstrates the proposed CNN structure and the hyperparameter tuning process through Bayesian optimization technique. The following subsection explains the Bayesian approach for hyperparameter optimization.

##### Bayesian Optimization Algorithm

Bayesian optimization is an effective approach for tackling global optimization problems. Global optimization addresses the challenge of determining an input that minimizes or maximizes the cost of a specific objective function. Most objective functions are non-convex, nonlinear, high-dimensional, noisy, and computationally expensive, making them challenging to evaluate [58]. Bayesian optimization creates a probabilistic model of the objective function *F*(*x*), known as the surrogate function, which is then efficiently searched with an acquisition function. Bayes Theorem is the base of the Bayesian optimization approach. Bayes Theorem calculates the conditional probability of an event A given another event B, P(A|B), as follows [58]

P(A|B) = P(B|A) ∗ P(A)/P(B)(1)

For optimization problems, Bayes theorem can be modified by omitting the marginal probability P(B) from the conditional probability equation [52] as follows

P(A|B) = P(B|A) ∗ P(A)(2)

The posterior probability is commonly referred to as the conditional probability P(A|B); the likelihood is referred to as the reverse conditional probability P(A|B); and the marginal probability P(A) is referred to as the prior probability [58]. As a result, an updated version of Bayes theorem can be written as

Posterior = Likelihood ∗ Prior(3)

The posterior probability is a function that approximates the objective function and is used to drive future search space sampling. The search space in our problem is the CNN hyperparameters. Bayesian optimization, as an informed search method, is distinguished by the use of an acquisition function that uses the posterior to sample the search space and pick the next sample for objective function evaluation. The optimization algorithm starts by using a probabilistic model for the objective function (the Surrogate function). The objective function in this study is represented by a Gaussian process model with a Matern 5/2 kernel. To begin, random samples from the search space$\;\left(x_{1},\;x_{2},\;\ldots ,\;x_{n}\right)$ are utilized to determine the objective function evaluation $F\left(x_{i}\right)$ at this sample. The samples and their assessments are collected in a sequential manner, resulting in a set of data points$\;S=\left\{x_{i},\;F\left(x_{i}\right),\;\ldots \;x_{n},\;F\left(x_{n}\right)\right\}$, where n is the number of samples. The set S is used to define the prior and likelihood function. The likelihood function is defined as the probability of observing the data given the objective function [58] as given in Equation (4).

(4) P(F|S)=P(S|F) * P(S)

After the prior and likelihood have been evaluated, the posterior is updated. The acquisition function, C, is then optimized over the Gaussian process surrogate function to select the next sample $x_{n}$, which is provided in Equation (5) [59].

(5) $$x_{n}={\mathrm{argmax}}_{x\;\;}C(x|S_{1:\;n-1})$$

The acquisition function is implemented in this study using the Expected Improvement algorithm as follows [59]

(6) $$C\left(x\right)=E\left[\max\left(F\left(x\right)-F\left(x^{+}\right),0\right)\right]\;$$

where E is the expectation operator, $F\left(x^{+}\right)$ is the objective function value for the best sample, and $x^{+}$ is the best sample position in the search space. The selected sample is then evaluated using the objective function, and the cycle is continued until the objective function reaches its minimum or the least objective is identified within the given run time. In this work, the objective function is evaluated for a maximum of 30 times as commonly recommended [60]; each evaluation is executed in single optimization iteration. A stopping criterion is applied if the observed objective passes a threshold value.

##### Optimization Variables

In this work, four variables are optimized using Bayesian optimization. These variables are

1. Initial Learning Rate

The learning rate (σ) is a CNN hyperparameter that controls how network weights are tuned in relation to the gradient descent cost. It controls how quickly the network learns. A global initial learning rate is set at the beginning of each optimization iteration. After a set number of training epochs, this value is steadily reduced. This method aids in the convergence of network parameters to the loss function minimum and shortening the training time. The initial learning rate is reduced piecewise in this study, with every 40 epochs, σ is reduced by a factor of 0.1. Throughout the optimization iterations, the values of the global initial learning rate cover the range (10^−2^–1).

2. Convolutional Block Depth (CBD)

The architecture of the proposed CNN is composed of three convolutional blocks. Each convolutional block is constructed by concatenating a number of basic blocks. The basic block consists of a convolution layer, batch normalization layer, and a ReLu layer. The number of basic blocks in each block equals to CBD, which is variable throughout the optimization process. The CBD is optimized over the range [1,2,3,4,5,6], hence multiple shallow and deep networks are examined in search of the ideal network architecture. The total number of convolutional layers is 3* CBD. Padding is added to each convolutional layer to make the spatial output size equal to the input size. Furthermore, the number of convolutional filters is set to be proportional to 1$/\sqrt{\mathrm{CBD}}$ to keep the amount of network parameters and computational load consistent for different CBD values.

3. Stochastic Gradient Descent Momentum

In this study, the introduced deep network is trained using the stochastic gradient descent with momentum (SGDM) optimizer. The SGDM is a version of the classic gradient descent technique in which a mini-batch subset of the training set is used to evaluate the gradient and update the parameters [61]. In a single epoch, the SGDM processes the complete training set using mini-batches. Due to the usage of mini-batches, the SGDM’s parameter updates are noisy. Consequently, the SGDM’s decline towards the loss function minimum is oscillatory. As shown in Equation (7), a momentum term (M) is introduced to the parameter update equation of SGDM to mitigate this tendency [61]. The momentum component adds a contribution of the previous iteration’s gradient into the current update, hence assisting in the smoothing of parameter updates.

(7) $$\beta_{i+1}=\beta_{i}\;–\;\sigma \;\nabla L\left(\beta_{i}\right)+M\;\left(\beta_{i}-\beta_{i-1}\right)$$

where β is the parameter vector which contains network neurons’ weights and biases, ∇L(β) is the loss function, L(β), gradient, σ is the learning rate, and i is the iteration number. In this work, the mini-batch size is set to 128, the maximum number of epochs is set to 150, and the momentum (M*)* is optimized over the range (0.75–0.99).

1. Regularization Coefficient

A decay term is introduced to the loss function of the SGDM to regularize the decay in the weights of network neurons in order to reduce overfitting [62]. This term is referred to as the Regularization Coefficient, ε, as depicted in Equation (8), which represents the regularized loss function $L_{r}$ [62].

(8) $$L_{r}\left(\beta \right)=L\left(\beta \right)+\varepsilon \;\Omega \left(w\right)$$

where w is the weight vector of network neurons, and Ω(w) is the regularization function given as $\Omega \left(w\right)=\frac{1}{2}wTw$. T is the matrix transpose operator [62].

#### 3.2.4. Optimal Model Selection and Testing

Bayes optimizer selects the optimal network hyperparameters by minimizing an objective function. In this study, the objective function is set as the classification error rate on the validation set, $\;E_{val}\;$, as given in Equation (9).

(9) $$E_{val}=1-\bar{PVS}$$

where $\bar{PVS}\;$is the mean prediction on the validation set. Multiple objective function evaluations, namely 30 evaluations, are performed for the purpose of optimizing the hyperparameters of the proposed CNN. In each evaluation, network hyperparameters are specified using the optimization variables, the network is trained, and classification error on the validation set is calculated. Without exposing the network to the test set, the optimal model is selected based on the lowest value of the validation set’s classification error. The best model is then assessed on a separate test set. 

The classifier’s ability to correctly detect the presence or absence of disease is crucial in medical applications. Therefore, the sensitivity (SN) and specificity (SP) performance metrics are used in this investigation to assess the generalization of the model on new data. A classifier’s sensitivity indicates its capacity to accurately identify diseased images, while the specificity indicates its ability to correctly identify normal images [63]. Furthermore, as we have nearly the same number of images for the normal and diseased classes, we considered the accuracy (AC) as well as an indicative performance measure of the proposed model. Equations for SN, SP, and AC are given as follows [63]

(10) $$\mathrm{SN}=\frac{\mathrm{TP}}{\mathrm{TP}+\mathrm{FN}}$$

(11) $$\mathrm{SP}=\frac{\mathrm{TN}}{\mathrm{TN}+\mathrm{FP}}$$

(12) $$\mathrm{AC}=\frac{\mathrm{TP}+\mathrm{TN}}{\mathrm{TN}+\mathrm{TP}+\mathrm{FN}+\mathrm{FP}}$$

where FN is the number of false negatives, FP is the number of false positives, TN is the number of true negatives and, TP is the number of true positives.

## 4. Results

The present study was implemented by computer codes created in MATLAB. Experiments were conducted on an Intel^®^ Xeon^®^ E-2286M CPU @ 2.40 GHz with 32 GB of RAM, single GPU, and Windows 10 Pro 64-bit.

Based on the introduced framework, the leukemia dataset was preprocessed and split into three subsets: 80% for training, 10% for validation, and 10% for testing. Each of the RGB channels of the augmented dataset was resized to [64 × 64] pixels. Afterwards the ranges of the optimization hyperparameters were set. The ranges of the initial learning rate, CBD, regularization coefficient, and SGDM momentum as well as the search functions were used to set variables over the optimization iterations are depicted in Table 1. Initial learning rate and momentum were searched on a logarithmic function scale. 

The SGDM algorithm was used to train the proposed network using the training subset with a mini-batch size of 128 images, piecewise drop rate for the learning rate of 0.1 per 40 epochs and mean and variance batch normalization decay rates of 0.1. Dropout approach with a probability of 0.5 was adopted to avoid the occurrence of overfitting. The number of objective function evaluations was set to 30. However, a stopping condition was set if the objective function recorded a value of 10^−4^ or less. Each objective function evaluation was executed in 150 epochs. At the first optimization iteration, the optimization variables, hyperparameters, were set randomly within the specified ranges. The number of convolutional blocks of the proposed CNN was set accordingly, and the network was trained, evaluated, and the objective function was computed and recorded. The Bayesian optimizer then selected the next set of hyperparameters for the second iteration based on the acquisition function maximization. During the second iteration, the selected hyperparameters were used to set the structure of the CNN, train and evaluate the model, and compute the objective function. The Bayesian optimizer used the results of the previous iteration and decided the next set of hyperparameters. The process continued until reaching the predefined maximum number of iterations, 30 iterations, or meeting the stopping criterion. The model that achieved the least objective was considered the optimal model and tested on the test set. Table 2 presents the results of optimizing the proposed CNN hyperparameters based on the Bayesian technique. Table 2 depicts the iteration number, the observed objective function value, CBD, initial learning rate, SGDM momentum, and regularization coefficient. The best CNN model is indicated in a bold font in the table. The optimal model achieved a validation error of zero at the 13th iteration. 

**Figure 5:**
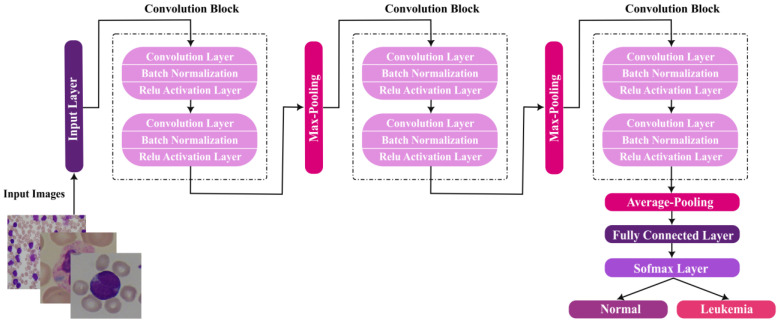
Architecture of the optimal Bayesian-based CNN model for ALL detection.

The architecture of the optimal CNN is composed of two basic blocks per convolutional block (i.e., CBD = 2). Subsequently, the optimal CNN is composed of six convolutional layers, six batch normalization layers, six ReLu layers, two average-pooling layers, one max-pooling layer, and a single softmax layer. Figure 5 shows the optimal structure of the proposed CNN. The hyperparameters of the optimal model is σ = 0.010006, ɛ = 8.5346 × 10^−10^, and M = 0.91561.

**Figure 6:**
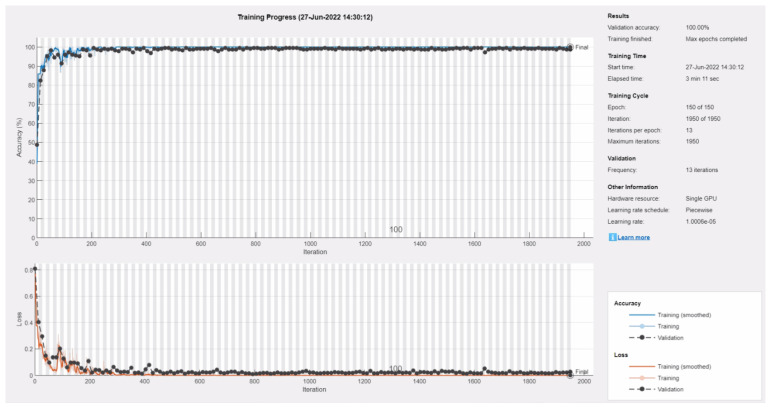
Training progress plot of the optimal proposed CNN model in the 13th optimization iteration. Upper subplot shows the percent accuracy for the training and validation subsets and lower subplot presents the corresponding loss.

Figure 6 presents the training progress plot of the proposed CNN’s optimal model. The upper subplot of Figure 6 shows the percent accuracy on the training and validation subsets. The lower subplot depicts the training and validation loss for the optimal model. This model achieved 100% validation accuracy after 150 epochs. As evidenced by the training progress plot, training and validation accuracy and loss curves behaved consistently and steadily after the network was learned till last epoch. Consequently, no overfitting was detected for this model.

**Figure 7:**
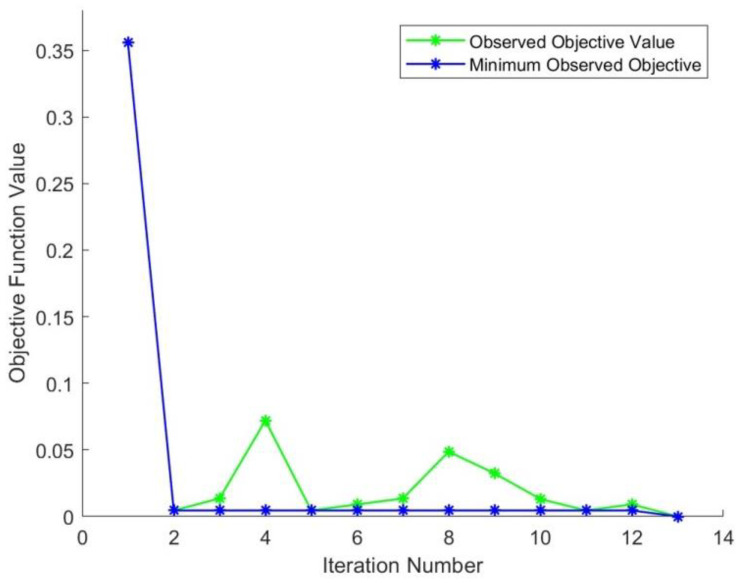
Objective function records versus the optimization iterations plot.

Figure 7 shows a plot that relates the observed objectives versus the optimization iterations. The minimum observed objective function values are also depicted on the same plot. The objective function values exhibit moderate fluctuation with a decreasing trend throughout the optimization procedure. 

**Figure 8:**
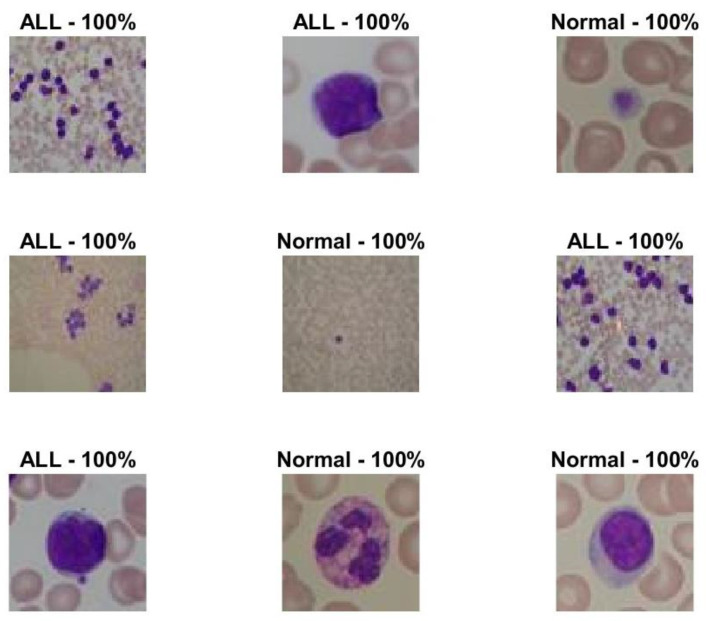
Sample test images along with their predicted classes and class probability. ALL denotes the diseased images.

The best model of the optimized CNN was assessed on the holdout test set. The optimal model achieved an accuracy, sensitivity, and specificity to detect ALL of 100% on the test set. Sample test images along with their predicted classes and class probability are depicted in Figure 8. The class probability of all presented images is 100% which reveals high confidence in the predictions made by the optimized model.

The classification performance of the optimal CNN model is compared to a non-optimized version of the same CNN to figure out the effectiveness of using the Bayesian optimization approach to fine-tune the model hyperparameters. Table 3 depicts the results of the optimized and non-optimized CNN models. The comparison is based on the classification error on the validation set, the run time of training and validating the CNN, and the classification accuracy on the validation set and test set. The hyperparameters of the non-optimized model is set to as: CBD = 3, σ = 0.0809, ε = 7.83 × 10^−3^, and M = 0.819. The comparison in Table 3 shows that the optimized model records higher accuracy than the non-optimized model for both validation and test sets and lower classification error as well. The run time of the optimized model is less than that of the non-optimized one. This could be related to the increased number of layers in the non-optimized model which requires more time for the increased number of parameters to learn. The comparison reveals the effectiveness of using Bayesian optimization to fine-tune the hyperparameters of CNN models in improving the classification performance.

The performance of the optimal model of the proposed ALL detection CNN is further assessed against a number of state-of-the-art deep learning ALL detectors. In this comparison, we compared the proposed CNN with optimized and non-optimized deep learning networks developed for the classification of leukemia. The studies selected for the comparison utilized either the entire ALL-IDB dataset or only one subset of it to train and test their models. Table 4 compares the optimal proposed ALL detection CNN with recent leukemia diagnosis CAD systems in terms of the used methodology, use of optimization, used dataset, and classification accuracy. The proposed CNN records higher classification accuracy than the non-optimized models in [8,22] and the Naïve Bayes classifier in [44]. It records an equal classification accuracy as the Decision Tree classifier of [44]. However, our Bayesian-based optimized CNN outperforms all optimized deep learning-based leukemia detectors in [53,54,55,56]. In general, the comparison reveals that the introduced Bayesian-based optimized CNN achieves superior classification performance over existing deep learning systems developed for ALL diagnosis. 

## 5. Conclusions

In this work, a new customized CNN is proposed for the detection of ALL in microscopic blood images. The architecture of the proposed CNN is set as an optimizable variable. The Bayesian optimization approach is utilized to determine the optimal architecture of the proposed CNN and optimize its hyperparameters. The Bayesian optimization method adopts an iterative procedure to minimize an objective function and select optimum network hyperparameters. Initial learning rate, SGDM algorithm momentum, regularization coefficient, and convolution block depth are the optimizable hyperparameters to be tuned by the Bayesian optimization method. The introduced CNN was trained and validated using a hybrid dataset which was formed by integrating the two publicly available ALL-IDB 1 and 2 datasets. To boost the classification performance of the proposed CNN, data augmentation through image transformations has been adopted to supplement the hybrid image set. The optimal CNN model selected by the Bayesian optimization method for ALL detection recorded accuracy, specificity, and sensitivity of 100% on a holdout test set. This high classification performance was obtained without the need to apply complicated image enhancement or segmentation methods on the input images. The optimized CNN model recorded higher classification accuracy than a non-optimized version of the proposed model which reveals the effectiveness of the Bayesian optimizer in fine-tuning the model hyperparameters. The optimized CNN introduced in this study outperforms the other optimized deep learning ALL classification models and strongly competes with state of the art alternatives. 

## Figures and Tables

**Table 1:** Optimization variables ranges and search functions used during the optimization iterations.

	Initial Learning Rate	CBD	Momentum	Regularization
Range	[10^−2^–1]	[1–6]	[0.75–0.99]	[10^−11^–10^−2^]
Search function	Logarithmic	-	-	Logarithmic

**Table 2:** Objective function records and corresponding hyperparameters estimates of the proposed CNN during the optimization iterations. Optimal model entries are presented in bold font.

Iteration	Objective Function	CBD	Initial Learning	Momentum	Regularization
1	0.35586	4	0.6922	0.90173	9.6527 × 10^−10^
2	0.0045045	2	0.075049	0.89149	4.9006 × 10^−5^
3	0.013514	6	0.042593	0.90022	5.1565 × 10^−7^
4	0.072072	1	0.098134	0.97037	4.6549 × 10^−5^
5	0.004504	5	0.078053	0.75109	9.4144 × 10^−6^
6	0.009009	1	0.071008	0.81437	2.1089 × 10^−10^
7	0.013514	3	0.080948	0.81932	7.8381 × 10^−3^
8	0.048649	3	0.15034	0.92367	9.1491 × 10^−5^
9	0.032432	4	0.051939	0.95414	4.125 × 10^−8^
10	0.013145	1	0.021743	0.98634	1.5784 × 10^−6^
11	0.004505	6	0.019914	0.86191	1.495 × 10^−3^
12	0.00908	2	0.07659	0.75475	8.1832 × 10^−5^
**13**	**0**	**2**	**0. 010006**	**0. 91561**	**8.5346 × 10^−10^**

**Table 3:** Comparison results of the proposed Bayesian-based optimal model with a non-optimized version of the proposed CNN.

	Classification Error on Validation Set	Validation Accuracy	Test Accuracy	Run Time (Sec)
Non-optimized Model	0.013514	98.65%	98.3%	248.5
Optimized Model	0	100%	100%	198.33

**Table 4:** Comparison results of the proposed Bayesian-based optimal CNN with the state-of the-art Leukemia detection systems. The accuracy is presented as percentage.

Methodology	Classifier	Optimization	Dataset	AC	Paper
Features extraction by AlexNet, CaffeNet, and VGG-f then feature fusion and selection by Gain Ratio algorithm.	SVM	-	ALL-IDB	99.2	[8]
Features extraction by AlexNet, GoogleNet, and SqueezeNet followed by feature fusion.	SVM	-	ALL-IDB 2	98.2	[22]
Features extraction by DarkNet and ShuffleNet followed by feature fusion and selection by Principal Component Analysis	Decision Tree	-	ALL-IDB	100	[44]
	Naïve Bayes	-		96	
Feature extraction by VGGNet. Optimal features are selected by a bio-inspired optimizer.	KNN, SVM, Decision Tree, Naive Bayes	Salp Swarm Optimization	ALL-IDB 2	96.1	[53]
Hand crafted features from input images and optimal feature selection by an optimizer.	Ensemble of classical ML classifiers	Social Spider Optimization	ALL-IDB 2	95.2	[54]
Image segmentation using Sparse Fuzzy C-Means clustering and optimized CNN for classification.	Customized CNN	Grey wolf-based Jaya Optimization	ALL-IDB 2	93.5	[55]
Attention-based Long-Short Term Memory for classification after feature selection.	ABiLSTM	Competitive Swarm Optimization	ALL-IDB 1	96	[56]
Bayesian-optimized CNN for classification	Customized CNN (BO-ALLCNN)	Bayesian Optimization	ALL-IDB	100	Proposed study

## Data Availability

Data sharing is not applicable to this article as the authors used a publicly available dataset, whose details are included in the ‘Materials and Methods’ section of this article.
